# Identification and characterization of microRNAs in *Baylisascaris schroederi* of the giant panda

**DOI:** 10.1186/1756-3305-6-216

**Published:** 2013-07-24

**Authors:** Guang-Hui Zhao, Min-Jun Xu, Xing-Quan Zhu

**Affiliations:** 1 College of Veterinary Medicine, Northwest A&F University, Yangling, Shaanxi Province, 712100, People’s Republic of China; 2 State Key Laboratory of Veterinary Etiological Biology, Key Laboratory of Veterinary Parasitology of Gansu Province, Lanzhou Veterinary Research Institute, Chinese Academy of Agricultural Sciences, Lanzhou, Gansu Province, 730046, People’s Republic of China

**Keywords:** *Baylisascaris schroederi*, Giant panda, microRNA (miRNA), miRNA target

## Abstract

**Background:**

*Baylisascaris schroederi* is one of the most significant threats to the giant panda’s survival, responsible for half of the deaths reported from 2001 to 2005. MicroRNA (miRNA) has been identified as one of the key factors for gene regulations at the post-transcriptional level, and also considered as a potential control and treatment target against infectious diseases.

**Methods:**

The present study investigated the miRNA profile of *B. schroederi* via high throughput sequencing and real-time quantitative PCR.

**Results:**

A total of 18.07 million raw reads were obtained and 18.01 million were identified with high quality. By analysis of standard stem-loop structures, 108 miRNA candidates were predicted, including 60 known miRNAs and 48 novel ones. Target prediction revealed that the “chitinase” was the most abundant target with 483 sequences, and 263 targets were related to ovarian and egg development. The ribosomal protein related sequences occupied 449 sequences.

**Conclusions:**

Previous studies have shown that some parasites secrete chitinases for exsheathment and/or for penetrating the peritrophic matrix of the host. It therefore seems that *B. schroederi* may be effectively regulated by miRNAs for development, invasion, and reproduction. Given that chitinases have been identified as important biological control agents for pests, identification of microRNAs in *B. schroederi* of the giant panda would provide useful information for the development of biological control strategies and/or vaccines against *B. schroederi* infection in the giant panda.

## Background

The giant panda (*Ailuropoda melanoleuca*) is one of flag and rare species for wildlife conservation, with an estimate of about 1600 restricted to Qinling, Minshan, Qionglai, Daxiangling, Xiaoxiangling and Liangshan mountains in China [1-3]. The giant panda is famous for its cute appearance, important taxonomic status and valuable gene pool for genetic studies [4-7]. Unfortunately, it is also one of the most endangered species due to limited habitats, surprisingly extensive poaching, and parasitic and other infections [8-10].

The roundworms in the family Ascarididae are parasitic nematodes with great medical, veterinary and economic significance [11,12]. Within this family, *Baylisascaris* spp. could infect animals and humans, resulting in visceral larval migrants (VLM), ocular larva migrants (OLM) and even neural larva migrants (NLM) that is normally fatal to some wild animals [13]. Among them, *B. schroederi* is the most common parasite in wild and captive giant pandas, and the VLM caused by *B. schroederi* infection was identified as the most significant threat to the survival of the giant panda, responsible for half of the deaths from 2001 to 2005 [8,14].

MicroRNAs (miRNAs) are small non-coding RNAs with a length of 18–25 nt which have been identified in various plants, animals and virus. They play key regulatory functions for gene expression at the post-transcriptional level [15-18] and are considered as a potential treatment target against parasitic and other infectious diseases [19,20]. Therefore, identification and prediction of miRNAs in pathogenic agents have important implications for controlling their infection. In the present study, the miRNA expression profile of *B. schroederi* was investigated by high throughput sequencing technology and real-time quantitative PCR.

## Methods

### Ethics statement

The present study was performed strictly according to the Guidelines and Recommendations for the Care and Use of Laboratory Animals of the Ministry of Health, China, and the study protocol was reviewed and approved by the Research Ethics Committee of Northwest A&F University.

### Parasites

Adult female nematodes were collected from the faeces of the giant pandas after anthelmintic treatment in Shaanxi Rare Wildlife Rescue Breeding Research Center rescued from Qinling Mountains in Shaanxi province, China. Worms were incubated in physiological saline for 3 h at 37°C and then washed extensively to get rid of contamination from the host. Female adults were identified by morphology and further ascertained as *B. schroederi* by sequencing the first internal transcribed spacer (ITS-1) of nuclear ribosomal DNA [21]. The parasites were then stored in liquid nitrogen for further study.

### Isolation of total RNA and small RNA

Total RNA and small RNA of the worms were prepared as described previously [22]. Briefly, one whole worm was grounded into fine powder under liquid nitrogen. The total RNA was prepared using TRIzol Reagent according to the manufacturer’s protocol (Invitrogen, USA). Ten micrograms of total RNA were used to separate small RNA of 20–40 nt length via a Novex 15% TBE-Urea gel. The purified fragments were ligated with 5′ and 3′ adaptors (Illumina, USA), re-purified on a Novex 10% TBE-Urea gel, and finally reversely transcribed with an RT-PCR kit. All the gels and kits were purchased from Invitrogen Co. Ltd.

### High-throughput sequencing and data analysis

The total RNA was deeply sequenced with Illumina Hiseq 2000 sequencer at HuaDa Genomic Co. Ltd, Shenzhen, China. The data was analyzed as described previously [23,24]. After base-calling, adaptors, reads smaller than 18 nt and those with low qualities were discarded, the remaining sequences were firstly searched against the Rfam databases (http://rfam.sanger.ac.uk/) to identify non-coding RNA, including rRNA, tRNA, snRNA, and snoRNA, and then searched against RepeatMasker (http://www.repeatmasker.org) to identify kinds and numbers of repetitive sequences. The genome sequence of *Ascaris suum* was used as a reference [25] and mapped with filtered reads via SOAP [26]. The miRNA precursors were identified with Mfold (http://www.bioinfo.rpi.edu/applications/mfold) and checked manually. Only the miRNAs with standard stem-loop structure and energy lower than −18 kcal/mol were saved to form the miRNA expression profile. The predicted mature miRNAs were mapped with *A. suum* miRNAs deposited in Sanger miRBase (http://www.mirbase.org/) to identify the known miRNAs and novel miRNAs.

The mRNA and EST sequences were downloaded from NCBI and used for target analysis of *B. schroederi* specific miRNAs via RNAhybrid [27]. The predicated targets were then performed for functional analysis via Gene Ontology (http://www.geneontology.org/) and DAVID database (http://david.abcc.ncifcrf.gov/).

### Analysis of miRNA expression level

The modified stem-loop real-time RT-PCR (ABI PRISM® 7300 Sequence Detection System) was used to determine the expressional level of representative miRNAs as described previously [28]. All reactions were carried out in triplicate with a 20 μl reaction mixture. The β-actin gene was used as the endogenous control with primers as follows: forward primer (5′-CTCGAAACAAGAATACGATG-3′) and reverse primer (5′-ACATGTGCCGTTGTATGATG-3′) [29]. Primers were synthesized by Shenggong Co, Ltd., Shanghai, China. The amplification cycles were as follows: 94°C for 32 s, 52°C for 30 s, 72°C for 30 s for 30 cycles. The expression level of miRNA was calculated as: N = 2^-ΔCt^, ΔCt = Ct_miRNA_-Ct_acin_[30].

## Results

### Profile characteristics of short RNAs

High-throughput sequencing generated 18.07 million raw reads. After base-calling, a total of 18.01 million reads showed high quality. After removing adaptors, poly-A and reads smaller than 18 nt, there were 16.26 million reads left for further analysis. The small non-coding RNAs, including rRNA, tRNA, snRNA and snoRNA, occupied 14.91% of total reads and 5.17% of unique reads, representing high redundancy of these non-coding RNAs. The percentages of rRNA were 13.48% and 4.41% in total and unique reads, respectively. Two types of repeat sequences were identified, namely rRNA: 0 (214 reads) and rRNA: 1 (3758 reads).

### Analysis of miRNA profile

**Figure 1:**
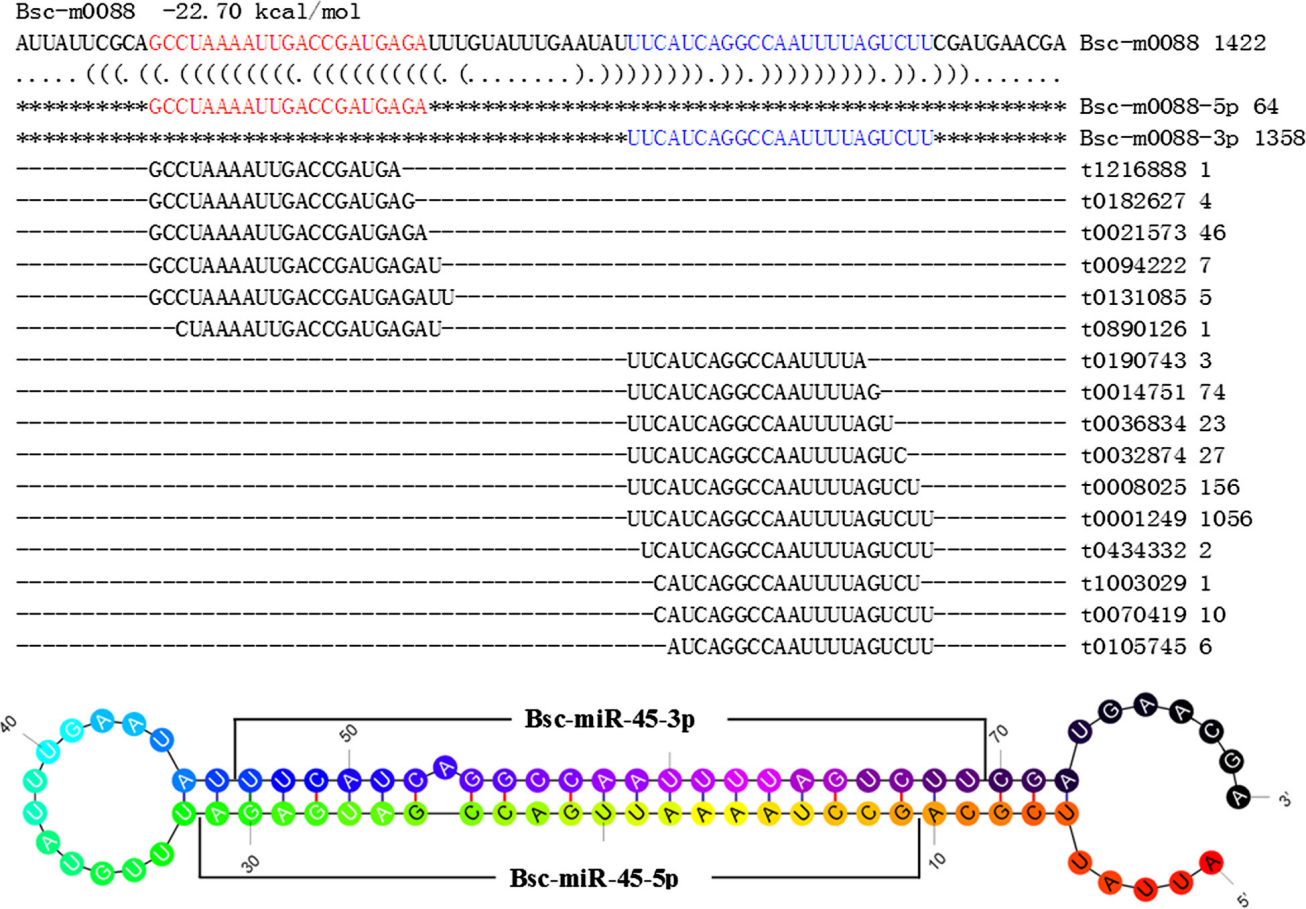
**Standard stem-loop structure of Bsc-miR-45.** First line: name of precursor and energy of stem-loop structure; Others: precursor sequences, mapped reads and stem-loop structure. Color of stem-loop structure: from red to black shows the direction of 5′ to 3′.

Mapping with genomic sequences of *A. suum*, we obtained 108 miRNA candidates with standard stem-loop structures. For 23 of these miRNAs, multiple locations were detected in the reference genome. Within them, 14 locations were found for the PAA-novel-44 (AGAAGCTGCGTTTTAAATGG), with 10 and 4 in the sense and antisense strands, respectively. The mature miRNA of PAA-novel-44 (Bsc-miR-44) was located at the 5′ arm of its precursors. The other significant miRNA was PAA-novel-45 (Bsc-miR-45), for which 8 locations were found with mature miRNAs at both the 5′ and 3′ arms of the precursor (Figure 1).

Compared with known *A. suum* miRNA deposited in the Sanger miRBase, 60 miRNAs were previously identified and 48 were novel candidates (see Additional file 1). The mature miRNAs at both 5′ and 3′ arms were identified for 58.3% (35/60) of known miRNAs and 8.3% (4/48) of the novel miRNAs.

### Target prediction and functional analysis

The 48 novel miRNAs of *B. schroederi* were further analyzed for target and function predictions. A total of 58438 mRNA/EST of *A. suum* (56616 sequences) and *A. lumbricoides* (1822 sequences), two nematodes being proposed as the same species with different genotypes [31], were downloaded from GenBank.

A total of 6975 targets (137 in average) were predicted for 48 *B. schroederi* novel miRNAs. Among these targets, 4 117 were well annotated and yielded a 1020 non-repeated dataset (see Additional file 2). The highest number of targets (853) was found for the Bsc-miR-51, but only one target was matched with miRNAs of Bsc-miR-21, Bsc-miR-34 and Bsc-miR-47. For well annotated targets, the most abundant target was the “chitinase” in *A. suum*, with 483 sequences. The target of “immunosuppressive ovarian message protein” was found within 199 sequences. Two other ovarian related targets were also predicted, namely “vitellogenin precursor-like protein” (33 sequences) and “ovarian abundant message protein” (31 sequences). Therefore, a total of 263 targets were related to ovarian and egg development.

In addition, the ribosomal protein related sequences occupied 449 sequences, including 152 sequences for 40S ribosomal proteins and 228 sequences for 60S ribosomal proteins, such as 40S ribosomal protein s2, 60S ribosomal protein l10, and 60S ribosomal protein l44. For parasite distributions of the annotated sequences, most of them were obtained from nematodes, including *Loa loa*, *Wuchereria bancrofti*, *Brugia malayi*, *Ascaris suum*, *A. lumbricoides* and *Haemonchus contortus*.

### miRNAs quantification

**Figure 2:**
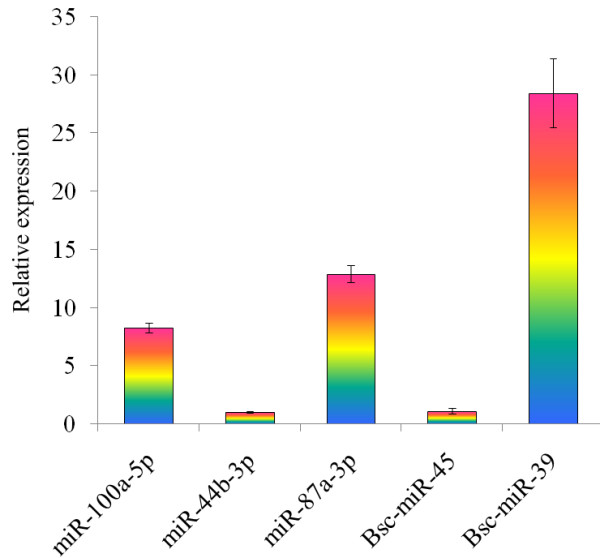
**Real-time quantitative PCR analysis for the three known miRNAs as well as three novel miRNAs of *B. schroederi*.** Some miRNAs had significant higher relative expression level than others, such as Bsc-miR-39, miR-87a-3p, and miR-100a-5p. The Bsc-miR-40 had a very low expression level and is not shown in the Figure.

Three known miRNAs named miR-100a-5p, miR-44b-3p, and miR-87a-3p, as well as three novel miRNAs of *B. schroederi* named Bsc-miR-45, Bsc-miR-39 and Bsc-miR-40, were randomly selected for real-time quantitative PCR analysis. All six miRNAs were successfully amplified (Figure 2). The relative expression levels of Bsc-miR-39 (28.43 ± 2.97), miR-87a-3p (12.89 ± 0.72), and miR-100a-5p (8.27 ± 0.43) were significantly higher than that of other miRNAs, while the relative expression levels of miR-44b-3p (1 ± 0.06) and Bsc-miR-45 (1.11 ± 0.23) were similar to that of the endogenous control. Very low expression level (0.03 ± 0) was detected for Bsc-miR-40, which was not shown in Figure 2.

## Discussion

The present study characterized the expression profile of miRNAs in *B. schroederi* from the giant panda in China. We predicted 108 miRNAs for *B. schroederi*, and this number was similar to that of miRNAs in *A. suum* predicted by Wang *et al.* (2011) [32]. However, the number of identified miRNAs herein was much smaller than that for *A. suum* in our previous study [23]. Given that the functions of miRNAs are mainly in regulating gene expression and ensuring genome maintenance, the expression level of miRNAs is highly sensitive to environmental and developmental signals [33-37]. Therefore, the miRNAs expression level of different parasite species, even the same species or stage, might be reflected by these conditions and infection status.

The regulating functions of miRNAs were played via complementing with target mRNAs to repress expression at protein translation level or affecting target mRNA maintenance. Target prediction for miRNAs of *B. schroederi* showed that the most abundant sequences were chitinases. The chitinases are enzymes widely distributed in bacteria, virus, fungi, plants and animals [38-40]. Some of the chitinases served non-redundant functions and were essential for survival, molting or development, digestion, molting, defense and pathogenicity of insects [41,42]. Some parasites, such as *Brugia malayi* and *Plasmodium*, can secrete chitinases for exsheathment of microfilaria and/or for penetrating the peritrophic matrix of host [43,44]. Recently, chitinases have been used to biologically control insect pests on transgenic plants either alone or in combination with other insecticidal proteins, and some chitinases also prove to be possibly useful as biocontrol agents and/or as vaccines [42]. Therefore, predicting microRNA targets would provide useful information for development of biological control strategies and/or vaccines against *B. schroederi* infection.

The other types of significant targets were proteins related to ovarian or egg development, including “immunosuppressive ovarian message protein” (199 sequences), “vitellogenin precursor-like protein” (33 sequences) and “ovarian abundant message protein” (31 sequences). For adult female *B. schroederi*, high numbers of ovarian related targets indicated the importance of miRNAs in regulating germ cell development of this nematode.

Ribosomes play a basic housekeeping role in global translation, and some rare developmental phenotypes could be detected in a number of ribosomal-protein-defective mutants [45]. Recent study also showed that the ribosome was emerging as a central hub in sensing the nature of the nascent protein chain, recruiting protein folding and translocation components, and integrating mRNA and nascent chain quality control [46]. In the present study, we predicted redundant high numbers of ribosome related targets (including 40S and 60S ribosomal proteins) complementing with *B. schroederi* novel miRNAs, indicating active activities and effective regulations of protein expression for this parasite.

## Conclusions

We firstly investigated the miRNA profile of *B. schroederi* from the giant panda in China. A total of 108 miRNA candidates were identified, with 48 representing novel miRNAs. Target analysis showed that high redundant numbers of sequences were related to chitinases, ovarian and ribosomes. These results provided novel resources for in-depth understanding of the biology of *B. schroederi*, and have profound implications for the development of miRNA-based drugs/vaccines against *B. schroederi* infection in the giant panda.

## Competing interests

The authors declare that they have no competing interests.

## Authors’ contributions

XQZ and GHZ conceived and designed the study, and critically revised the manuscript. GHZ and MJX performed the experiments, analyzed the data and drafted the manuscript. All authors read and approved the final manuscript.

## Supplementary Material

Additional file 1 **Known and novel miRNAs of *Baylisascaris schroederi*.** This Excel table describes in detail the sequences of the identified known (n = 60) and novel (n = 48) miRNAs from *B. schroederi*. Click here for file

Additional file 2 **Well annotated targets of the 48 novel/specific miRNAs of *Baylisascaris schroederi*.** This Excel table details the predicted targets and their origins of the 48 novel/specific miRNAs identified from *B. schroederi*. Click here for file
